# Large-scale wearable data reveal digital phenotypes for daily-life stress detection

**DOI:** 10.1038/s41746-018-0074-9

**Published:** 2018-12-12

**Authors:** Elena Smets, Emmanuel Rios Velazquez, Giuseppina Schiavone, Imen Chakroun, Ellie D’Hondt, Walter De Raedt, Jan Cornelis, Olivier Janssens, Sofie Van Hoecke, Stephan Claes, Ilse Van Diest, Chris Van Hoof

**Affiliations:** 1 Electrical Engineering-ESAT, KU Leuven, Leuven, Belgium; 2 Imec, Heverlee, Belgium; 3 Imec, Holst Centre, Eindhoven, The Netherlands; 4 IDLab, Ghent University—imec, Ghent, Belgium; 5 University Psychiatric Center & Department of Neurosciences, KU Leuven, Leuven, Belgium; 6 Faculty of Psychology and Educational Sciences, KU Leuven, Leuven, Belgium

**Keywords:** Predictive markers, Preventive medicine

## Abstract

Physiological signals have shown to be reliable indicators of stress in laboratory studies, yet large-scale ambulatory validation is lacking. We present a large-scale cross-sectional study for ambulatory stress detection, consisting of 1002 subjects, containing subjects’ demographics, baseline psychological information, and five consecutive days of free-living physiological and contextual measurements, collected through wearable devices and smartphones. This dataset represents a healthy population, showing associations between wearable physiological signals and self-reported daily-life stress. Using a data-driven approach, we identified digital phenotypes characterized by self-reported poor health indicators and high depression, anxiety and stress scores that are associated with blunted physiological responses to stress. These results emphasize the need for large-scale collections of multi-sensor data, to build personalized stress models for precision medicine.

## Introduction

Since Hans Selye’s definition of stress as “the nonspecific response of the body to any demand”,^1^ many studies have revealed the negative influence of an overload of stress on health and wellbeing. Observational data suggest associations between psychological stress and depression, cardiovascular disease, sudden death, and myocardial infarction.^2,3^ Early detection and prevention of the adverse consequences of stress are therefore of utmost importance, and require personalized prevention and treatment strategies that take individual variability into account, as is suggested in the Precision Medicine Initiative.^4^

Towards precision medicine, digital phenotypes are a new paradigm to extend our assessment of human illness beyond traditional examinations.^5^ They represent a subject’s interactions with digital technologies such as connected health devices and smartphones to generate longitudinal, individual health profiles. Leveraging data-driven approaches, these data can fundamentally change our understanding of disease prognoses and provide new insights towards disease prevention and early detection.^5^

The most widely used method and current gold-standard to assess stress is by means of questionnaires, e.g., the Perceived Stress Scale (PSS).^6^ However, these questionnaires are qualitative, time-consuming, and reflect responses collected during spot-checks only. This limits the accurate monitoring of stress and the use of just-in-time interventions to reduce stress. Therefore, research has focused on finding continuous and quantitative physiological markers of stress,^7^ by exploiting measurable functioning of the sympathetic nervous system’s fight-or-flight response,^8^ such as skin conductance (SC), the electrocardiogram (ECG), the electromyogram (EMG), blood pressure (BP), and skin temperature (ST).^9^ These have shown to be reliable indicators of stress in laboratory conditions.^9^

In recent years, the growing availability of wearable sensors has led to increased research towards continuous, ambulatory monitoring of stress. So far mainly small-scale studies of 20–50 subjects have been conducted.^10–12^ Multiple findings suggest that physiological responses to stress tend to be person-dependent.^13,14^ Therefore, large datasets are essential to grasp subject-to-subject variability and develop personalized risk profiles. Furthermore, in the majority of ambulatory trials, subjects’ demographics and psychological baseline profiles (e.g., self-reported anxiety and depression levels) are either overlooked or not assessed.^10,11^ Context information is often not measured,^10,12^ although it can also provide actionable insights.^7,11^

Here we present the SWEET study (Stress in the Work EnvironmEnT): a comprehensive, cross-sectional study on an office workers’ population of 1002 healthy volunteers, who were monitored continuously for 5 consecutive days. We collected baseline psychological information, 5 consecutive days of free-living physiological data through wearables and smartphone-based contextual measurements, self-reported stress through ecological momentary assessments (EMAs) and physiological responses to an application-based stress test. We show strong associations between physiology, contextual information, and behavior, highlighting the benefit of multi-sensor information to improve our understanding of stress in daily life. We show that physiological signals differ significantly according to reported stress levels and identified stress digital phenotypes, characterized by self-reported poor health and high depression, anxiety and stress scores, that are associated with blunted physiological stress responses. Building on preventive health, these findings and this comprehensive dataset of physiology, context, and stress in the daily life, allow for multi-sensor models to detect stress in daily life.

## Results

### Dataset quality and compliance

A comprehensive summary of the intake questionnaire, including subject demographics and psychological baseline information is available in S1 Table. The measurements lasted 5 days per subject, starting on Thursday morning and ending on Monday evening. An overview of self-reported stress responses through EMAs and compliance is available in S2 Table. Subjects provided on average 25 self-reported stress responses, with highly imbalanced data, containing 50.4% no stress and only 0.3% extremely high stress, reported on a five-point Likert scale. Therefore, the three highest stress levels were merged, representing 14.3% of the data, so that three, instead of five, levels of stress (S1 = no stress, S2 = light stress, S3 = high stress) were considered.

An overview of smartphone and wearable sensor data quality is available in S3 Table and S4 Table, respectively. High quality physiological data (the chest patch, measuring ECG and acceleration (ACC), see Methods, had on average 86.4 ± 8.2% good quality data; the Chillband, measuring SC, ST, and ACC, had on average 96.3 ± 2.2% good quality data), was complemented with smartphone-based context information such as location and audio features (see Methods). Using high quality physiological signals, 18 features were calculated (6 ECG features, 8 SC features, and 4 ST features) in a window of 5 min, with 4 min overlap. An overview can be found in material and methods and S5 Table.

Further, we compared questionnaire-based lifestyle (e.g., practicing sports, smoking habits) and health indicators (e.g., sleep quality, depression levels). Our findings represent a large-scale verification supporting previous work and confirm the value of data sampled with validated questionnaires (S1 Fig. and S1 Text).

### Associations between physiology, context, and behavior

We investigated correlations between physiology, context, and behavior in order to improve our understanding of stress in daily life.

Through EMAs we daily asked questions related to stress, activity, food and beverage consumption, sleep quality, and gastro-intestinal symptoms. Based on fixed day and night intervals, circadian rhythms of the physiological signals can be identified, namely lower average HR during the night and higher SC and ST (mean HR_day_: 74.6 ± 12.7, mean HR_night_: 63.0 ± 10.2, Wilcoxon ranksum *p* < 0.001; mean SC_day_: 1.7 ± 2.7, mean SC_night_: 2.8 ± 3.4, Wilcoxon ranksum *p* < 0.001; mean ST_day_: 31.4 ± 2.1, mean ST_night_: 33.1 ± 2.6, Wilcoxon ranksum *p* < 0.001, day = 06 am–23:59 pm, night = 00–06 am). During weekdays (i.e., Thursday, Friday, and Monday) consumption of caffeinated beverages or breakfast corresponded to higher stress levels (caffeine: 1.84 ± 0.81, breakfast: 1.87 ± 0.81, average: 1.77 ± 0.83, Wilcoxon ranksum *p* < 0.001), while dinner or alcohol consumption, corresponded to lower stress levels (dinner: 1.51 ± 0.71, alcohol: 1.30 ± 0.64, average: 1.77 ± 0.83, Wilcoxon ranksum *p* < 0.001). During the weekend (i.e., Saturday and Sunday), the consumption of alcohol was associated with lower stress levels (alcohol: 1.34 ± 0.66, average: 1.45 ± 0.71, Wilcoxon ranksum *p* = 0.001), other reported consumptions did not show significant differences. A possible confounder here could be time of the day since breakfast is consumed most in the morning (82% of reports between 6 and 10 h), and alcohol and dinner most in the evening (alcohol: 61% of reports between 18 and 22 h, dinner: 65% of reports between 18 and 22 h), caffeine was reported equally throughout the day, but less during the evening (32% of reports between 6 and 10 h, 37% between 10 and 14 h, 23% between 14 and 18 h, and 7% between 18 and 22 h).

Further, linear mixed effects models were computed to investigate associations between repeated measures. A significant negative association between self-reported stress and self-reported pleasure (based on the Self Assessment Manikin (SAM)) was observed, with higher levels of self-reported stress corresponding to decreasing levels of pleasure (Table 1). It can be speculated that rating of high self-reported stress is likely associated with the feeling of distress (negative stress) rather than eustress (positive stress). The standard deviation of the magnitude of acceleration (ACC SD), was associated with intensity of movement as ACC SD was higher during self-reported high-intensity activities (low-intensity, i.e., lying, sitting, and standing: 0.0175 ± 0.0089, high-intensity, i.e., walking, running, biking, driving car, and other activities: 0.0189 ± 0.0096; Wilcoxon ranksum *p* < 0.001) and HR and SC features increased with ACC SD (Table 1) while ST decreased with ACC SD (Table 1). This illustrates that physical activity is associated with changes in physiology, highlighting the challenge of differentiating physiological changes caused by physical activity from those caused by stress.^12,15^ Therefore, to account for the confounding effect of physical activity on physiology and stress, we excluded segments of high activity in the subsequent analysis. Finally, increasing activity levels (ACC SD), decreased the quality of physiological signals (Table 1), an issue inherent to the free-living nature of the study.

**Table 1:** Effects of repeated measures

Formula	B ± SE	Inferential statistics		
		Test statistic and df	*p*-Value	Significance
Stress~Pleasure + (1 | subject)	−0.22 ± 0.01	*X*^2^(2,3) = 1353.5	<0.001	*
SC mean~ACC SD + (1 | subject)	0.93 ± 0.02	*X*^2^(3,4) = 2448.6	<0.001	*
HR mean~ACC SD + (1 | subject)	12.04 ± 0.02	*X*^2^(3,4) = 488,051	<0.001	*
ST mean~ACC SD + (1 | subject)	−5.61 ± 0.02	*X*^2^(3,4) = 87,378	<0.001	*
SC Quality~ACC SD + (1 | subject)	−0.23 ± 0.0008	*X*^2^(3,4) = 90,652	<0.001	*
ECG Quality~ACC SD + (1 | subject)	−0.33 ± 0.0009	*X*^2^(3,4) = 115,642	<0.001	*

Results of the linear mixed effects models for repeated measures. For each model, the fixed effect coefficient is presented with standard error (B ± SE) and inferential statistics on the significance of the effect, which were calculated by testing the change in model performance (based on Akaike’s information criterion) when a given predictor (e.g., pleasure) was excluded from the model using an ANOVA test

**Fig. 1:**
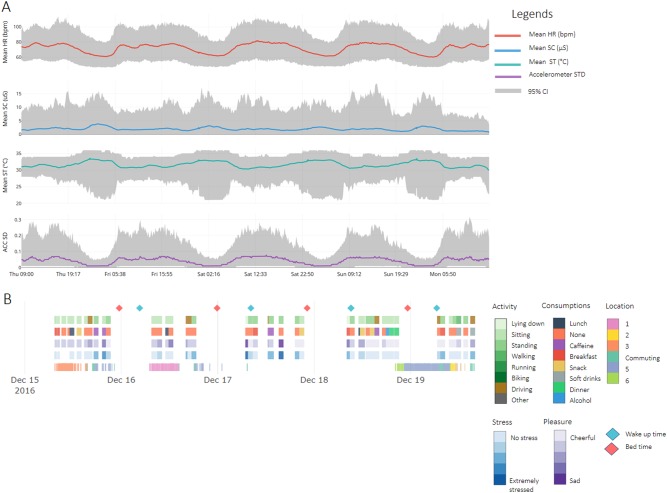
Physiology and context timeline. **a** Healthy-population physiological data over 5 days of measurements depicting smoothed daily profiles of high quality (Quality > 0.8) physiological signals (mean HR, SC, and ST) and activity (ACC SD), averaged in 1 min windows. **b** Self-reported annotations (stress, pleasure, activity, consumptions, and wake up/bed times) and location, as indicated with vertical lines when available for a representative subject. Location data are indicated as unique stay locations or commuting locations. An online version of **a** and **b** can be downloaded from https://drive.google.com/open?id=1Z1q0YLG8cvUllSM84X3mgwmCCBjfO0M7

Five days of measurements of physiological data and acceleration are shown in Fig. 1a, indicating daily variations in a healthy population. Behavior and mood self-annotations captured through EMA questionnaires and smart-phone sensor data are shown for a single representative subject in Fig. 1b.

### Associations between physiological signals and self-reported stress levels

We aim to use physiological patterns to develop models for psychophysiological stress detection. Therefore, we show here a variety of associations between physiological features and self-reported stress levels, in a healthy population. An overview of all physiological features calculated is presented in S5 Table.

**Fig. 2:**
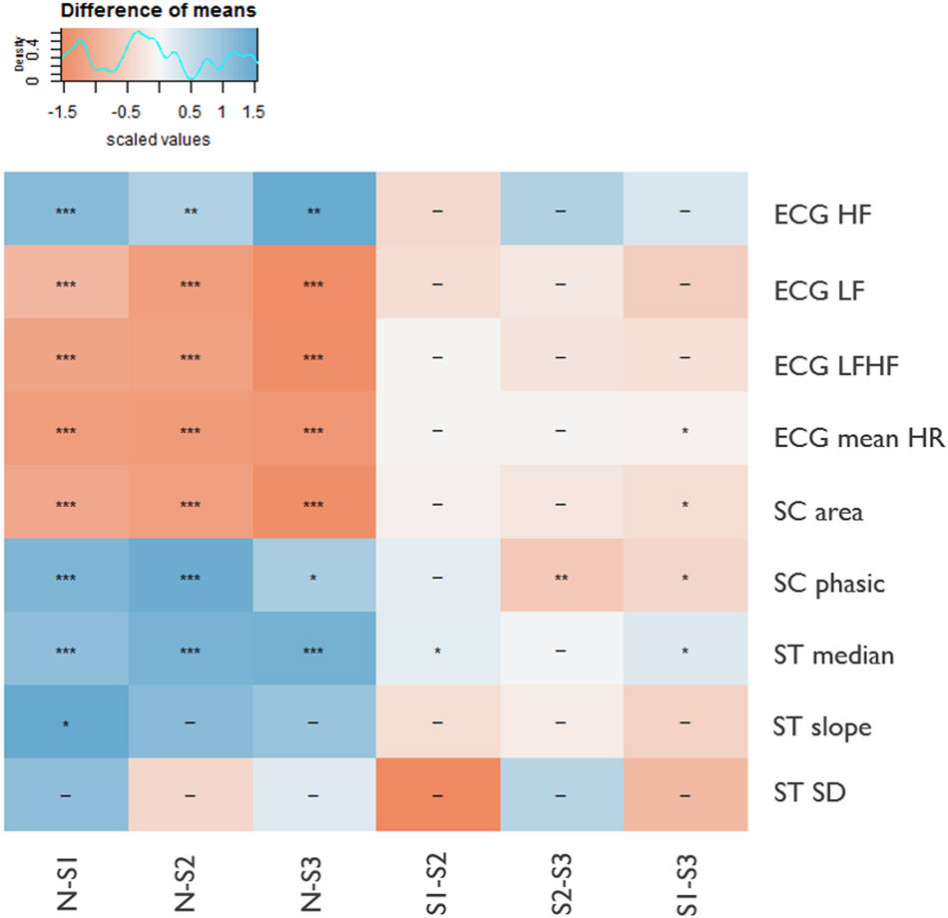
Associations between physiological features and self-reported stress levels. Each row represents a physiological feature, columns represent the difference of the median of normalized features during the night (N) (00–06 am) and stress levels (S1, S2, and S3). Colors indicate positive (blue) or negative (red) differences. For example, SC phasic is significantly higher (blue) during the night as compared to during all reported stress levels, and significantly lower (red) during S1 as compared to S2 and S3. Symbols: *p < 0.05, **p < 0.005, ***p < 0.0005

Mean differences of uncorrelated physiological features (*r* *<* 0.7), normalized per subject, across self-reported daytime stress levels (S1, S2, and S3) and nighttime (00–06 am, N), included as a baseline rest condition, are shown in Fig. 2. Population variations (averages and 95% CI) of physiological features across self-reported stress levels and nighttime are presented in Table 2, an extended version, including all physiological features, can be found in S6 Table.

**Table 2:** Physiological features across self-reported stress levels

	Night (mean, 95% CI)	S1 (mean, 95% CI)	S2 (mean, 95% CI)	S3 (mean, 95% CI)
ECG HF (×10^−3^)	0.69 (0.018, 3.6)	0.62 (0.016, 2.9)	0.66 (0.024, 2.8)	0.62 (0.023, 2.7)
ECG LF (×10^−3^)	1.1 (0.054, 5.0)	1.1 (0.074, 4.2)	1.1 (0.097, 4.0)	1.1 (0.1, 4.0)
ECG LFHF	3.5 (0.29,14.6)	3.8 (0.48, 15.0)	3.5 (0.51, 13.8)	3.7 (0.53, 14.4)
ECG mean HR	62.5 (47.3, 83.3)	72.1 (52.1,95.1)	73.4 (53.6, 95.5)	74.6 (56.0, 96.1)
SC area	0.71 (0, 6.2)	1.9 (0, 16.0)	1.7 (0, 14.5)	2.0 (0, 16.5)
SC phasic	18.3 (0, 157.5)	8.3 (0, 78.9)	7.7 (0, 72.5)	8.2 (0, 70.4)
ST median	32.9 (21.0, 36.0)	31.5 (27.0, 35.0)	31.2 (27.0, 34.0)	31.3 (28.0, 34.0)
ST slope (×10^−3^)	0.061 (−4.6, 4.7)	0.34 (-4.4, 4.9)	0.30 (-4.3, 4.9)	0.31 (−4.2, 4.9)
ST SD	0.10 (0, 0.50)	0.14 (0, 0.50)	0.13 (0, 0.50)	0.13 (0, 0.50)

Population mean and 95% confidence interval (CI) of physiological features during the night (00–06 am) and different stress levels (S1, S2, and S3).

For all the time instances, S1–S3 and N, only periods in which the activity level was lower than the empirical threshold (ACC SD < 0.04)^16^ and good quality (Quality > 0.8) data were considered to exclude artifacts and physiology variations due to physical activity. All features, except ST slope and ST SD (i.e., ST standard deviation, see S5 Table), were significantly different during nighttime (N) compared to during daytime self-reported stress levels (S1, S2, S3) (Fig. 2). ECG LF (i.e., the low frequency component of the RR signal, see S5 Table) and LFHF (i.e., the ratio of LF and HF), mean HR, SC area (i.e., the sum of the area of SC responses, see S5 Table) were lower at night. The ST median, SC phasic (i.e., power of the phasic SC component, see S5 Table), and ECG HF (i.e., the high frequency component of the RR signal, see S5 Table) were higher at night.

Additionally, mean HR was significantly lower in S1 as compared to S3. Mean HR has a strong negative correlation with RMSSD (i.e., root mean square of the successive RR differences, a time domain HRV feature, see S5 Table) (mean HR, RMSSD: *r* = −0.99), which is significantly higher in S1 as compared to S3 (see S6 Table). These results confirm findings in laboratory studies reporting an increase in HR and decrease in HRV with increasing stress levels.^17–19^ The frequency domain HRV features, i.e., the LF signal, HF signal and the ratio of LF and HF signals, did not change significantly during S1 as compared to S2 and S3 and during S2 as compared to S3. In literature, the HF component is thought to represent the cardiac parasympathetic nerve activity, which is active during rest conditions, and the LF component to represent the sympathetic system, which is active during stress conditions.^20^ The LF and LFHF components are therefore expected to be higher during stress conditions and the HF component lower.^20^ However, varying results have been reported in literature and in general RMSSD has been reported to be more reliable than LFHF,^20,21^ in particular because of the mechanical effects of respiration on HF power and the influence of the prevailing heart rate on LF power.^20^ Furthermore, SC area was lower in S1 compared to S3, as reported previously in ref. ^22^. SC phasic was lower in S2 compared to S3 and lower in S1 compared to S3, as expected based on previous laboratory research,^23,24^ indicating that higher stress levels are associated with higher power of the phasic SC component. Finally, the ST median was higher in S1 compared to S2 and S3, which indicates that ST amplitude decreases with stress.^25^ For most of the features no significant differences were found between S2 and S3. This could either indicate that in general subjects have difficulties in making distinctions between light and high stress levels or that physiological features cannot distinguish between these levels at a population level. Significant differences between S1 and S3, the two most extremes of the stress scale, were found for ST median, SC phasic, SC area, and ECG mean HR. A significant difference between S2 and S3 was found only for SC phasic and between S1 and S2 only for ST median.

Overall, the physiological signals measured in daily life showed significant differences between night and different stress levels, in line with previous findings of laboratory studies. These results confirm on a large scale the potential of physiological signals for detecting stress in daily life.

### Towards digital phenotypes for psychophysiological stress detection

We used a data-driven approach to uncover digital phenotypes of subjects’ daily life stress responses. We developed random forest models using a leave-one-subject-out cross-validation to link physiological features to self-reported stress. We used the classifiers’ performances to identify and characterize digital phenotypes representing subjects with similar psychological baseline, physiological responses to stress and health indicators.

Only good quality (Quality > 0.8) and low activity (ACC SD < 0.04) data were included for 568 subjects, with complete data (i.e., simultaneous continuous recording from wearables and EMAs). The remaining subjects had missing data in one of the two sensors or lacked mobile EMA data, and were not included in this analysis. To account for possible bias we compared baseline psychological questionnaires of the excluded subjects and found no significant difference with the included subjects (PSS included subjects: 14.2 ± 6.1, PSS excluded subjects: 14.6 ± 6.1, Wilcoxon ranksum *p* = 0.19). The classification performance, as calculated using the average F1-score across all subjects, was 0.43 (95% CI: 0.05–0.86), which is slightly better than the F1-score of 0.36, obtained when all samples are classified as the majority class (i.e., S1). The most important features across all subjects were ST median, SC phasic and SC diff2. An overview of feature importance is presented in S2 Fig.

Subjects were categorized in groups of low performance (*n* = 216), with F1-score < 0.33 (performance as good as random), medium performance (*n* = 249), with 0.33 < F1-score < 0.66 and high performance (*n* = 103), with F1-score > 0.66. We compared three aspects of each group: self-reported stress imbalance, physiological dynamic range and demographics, and psychological background information.

Subjects in the high performance group had on average a more imbalanced dataset (86% no stress, 12% light stress, and 2% high stress), compared to the low performance group (26% no stress, 45% light stress, and 29% high stress). This imbalance could provide an explanation for the difference between low and high performance. Additionally, on average subjects in the low performance group reported 26 times (SD_low_ = 14) their stress levels on the EMA’s, whereas for the medium and high performance groups subjects reported their stress levels on average 31 times (SD_medium_ = 13, SD_high_ = 12). Although the difference is small, the response rate for the low performance group was significantly lower (p < 0.001) as compared to the medium and high performance groups.

**Fig. 3:**
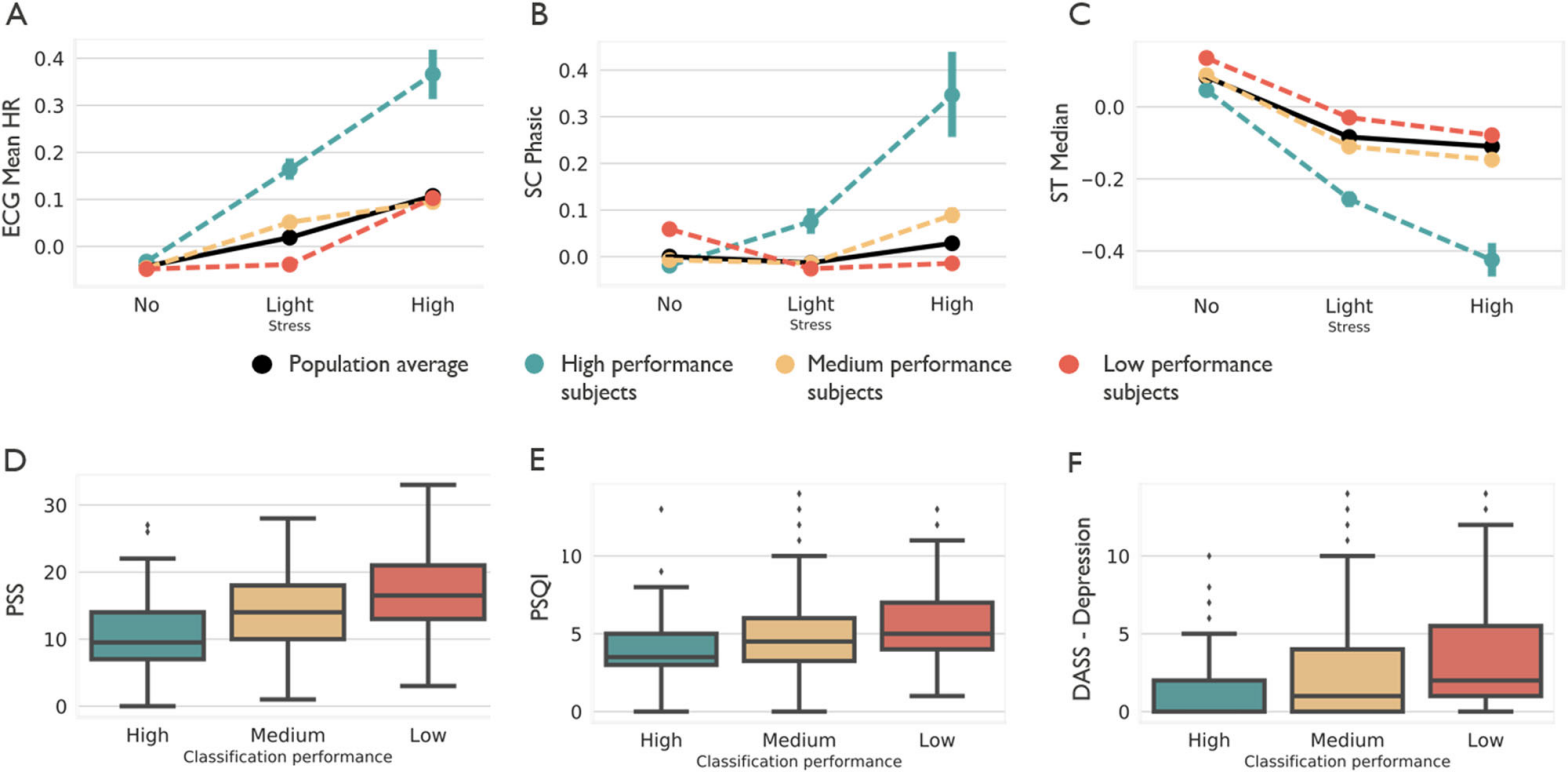
Comparison of subjects with low, medium and high classification performance. In **a**–**c** average features ECG mean HR, SC phasic, and ST median are shown respectively for low (red), medium (yellow), and high performance (green) groups and compared with the entire population average (black) in phases of no, light and high stress. In **d**–**f** baseline psychological information of subjects in low, medium, and high performance groups are compared

However, we also found that for 15 out of 18 features, the high performance group had a higher dynamic range (i.e., a larger average difference per physiological feature between low and high stress) as compared to the low performance group. In S2 Text we show that this effect is significantly different as compared to random assignment in three groups. Examples for mean HR, phasic SC, and median ST, are shown in Fig. 3a–c respectively; a complete summary for all features is provided in S3 Fig.

To account for possible confounders we further investigated subjects’ demographics and psychological information, based on the intake questionnaire, in the three groups. There was no difference in gender in all three groups (*X*^2^ low-high performance: *p* = 0.62, *X*^2^ low-medium performance: *p* = 0.41, *X*^2^ medium-high performance: *p* = 0.92). On average subjects in the high performance group reported a healthier lifestyle and lower baseline depression, anxiety and stress levels than subjects in the low performance group (Fig. 3d–f). They report to eat less take-out (low performance group: 1.1 ± 1.3 times per week, high performance group: 0.8 ± 0.9 times per week, Kruskal–Wallis *p* = 0.04), to practice more sports (low performance group: 26% does not practice sports, high performance group: 18% does not practice sports, *X*^2^ = 0.01), they have higher sleep quality based on the Pittsburgh Sleep Quality Index (PSQI scores higher than 5 indicate worse sleep quality; low performance group: 5.3 ± 2.5, high performance group: 4.1 ± 2.3, Kruskal–Wallis *p* < 0.001) and score lower on depression scale (Depression Anxiety Stress Scale (DASS)—depression scale; low performance group: 3.5 ± 3.4, high performance group: 1.4 ± 2.1, Kruskal–Wallis *p* < 0.001), anxiety scale (DASS—anxiety scale; low performance group: 2.6 ± 2.9, high performance group: 1.0 ± 1.7, Kruskal–Wallis *p* < 0.001) and stress scales (DASS—stress scale; low performance group: 6.5 ± 3.9, high performance group: 3.1 ± 3.2; PSS; low performance group: 17.1 ± 5.6, high performance group: 10.5 ± 5.5, Kruskal–Wallis *p* < 0.001) as compared to subjects in the low performance group. Subjects in the high performance group are also significantly older (low performance group: 38.6 ± 10.0, high performance group: 41.7 ± 10.0, Kruskal–Wallis *p* = 0.007).

## Discussion

To assess stress we collected a dataset of 1002 subjects during five consecutive days, including a wide variety of subject background information, physiological data in ambulatory settings and smartphone-based self-reports and contextual information. We found significant differences between physiological features for ECG, SC, and ST between different stress levels and nighttime baseline, confirming laboratory findings and indicating the potential of psychophysiological stress detection in daily life on a large-scale population.

Additionally, we compared digital phenotypes based on wearable and self-reported data emerging from a data-driven analysis. Although the classification performance of the generalized models (F1-score = 0.43) does not allow use in practice yet, we found that physiological responses to stress strongly differ among subjects, distinguishing groups with small and large dynamic ranges of the physiological features. These results highlight the need for future research to focus on personalized models as subjects differ in the magnitude and type of their physiological stress response. These groups are also characterized by different psychological baselines and demographics, where the group with a more blunted physiological stress-reactivity (small dynamic range) tend to report a less healthy lifestyle and higher depression, anxiety and stress scores than the more responsive group (large dynamic range). These findings suggest that self-reported poor health and high depression scores are negatively correlated to physiological reactivity. Similar findings have been reported previously in laboratory research,^26^ but to date no studies have investigated this relationship in real-life ambulatory physiological recordings. In the current study, a general sample of a healthy population was included, where ‘healthy’ was broadly defined as being able to go to work. Although several subjects scored high on the DASS or PSS scales, they were not diagnosed with any clinical disorder as per the DSM-V guidelines. The questionnaires merely provide a quantitative measure of distress along the axes of depression, anxiety and stress, not a categorical measure of clinical diagnoses. In future research, it would be interesting to compare the results of this baseline population with those of subjects with a clinical diagnosis, such as depression.

Although this dataset provides a wide variety of demographic profiles, caution for bias is needed when analyzing and interpreting the results. For example, subjects were mostly educated employees with sedentary jobs. It is possible that highly educated persons show different stress profiles compared with lower educated persons, or that highly educated persons would be better at wearing the wearable devices, which would bias the results. Also, to translate these results to persons with more active jobs or for example high performance athletes, additional experiments are needed.

Identifying the concept of stress in ambulant conditions is challenging as the gold standard is based on self-reports, which could lead to bias and reduced classification accuracies as compared to controlled laboratory studies in which stressors are artificially induced. This raises the question whether indeed stress is detected or rather an activation of the autonomic nervous system (ANS). From a data analytical perspective, one could argue stress is detected since the models are trained based on a, self-reported, stress reference, however this can be also biased due to subjective perception. From a psychophysiological perspective, it is not clear whether the physiological sensing models can differentiate between actual stress and arousal or an ANS activation. Further research should compare the link between physiological signals and self-reported stress-responses on the one hand and self-reported pleasure, arousal and control levels based on the SAM on the other hand to better differentiate stress and arousal levels.

This manuscript focused on the prediction of stress using physiological parameters. In the future, it could be investigated how context information (e.g., location, noise levels, ambient light), combined with physiological data, could be used to improve the performance of stress detection models. Further, in our study physiological data during high physical activity was excluded. It could be investigated if accelerometer data could be used to improve signal quality or to improve model performance, by incorporating the accelerometer signal itself for stress prediction and physical activity as an additional class. Additionally, the links between physiology, sleep quality and gastro-intestinal symptoms (Leuven Postprandial Distress Scale) for different psychological profiles (e.g., high versus low depression, high versus low stress), need to be investigated.

The results of this study provide a baseline for large-scale ambulatory population monitoring to uncover blunted physiological responses to stress. Furthermore, these findings have important implications related to stress modeling strategies, indicating that stress detection models should be tailored to phenotypes by including multi-sensor data sources, as subjects with different physiological responses to stress, display different health statuses. This study exemplifies how large-scale, data-driven analytics can be used to derive digital phenotypes and generate new insights into stress detection and disease interception in general. Continuous stress detection will form the basis to enable highly personalized, just-in-time interventions for preventive health.

## Methods

### Experiment

**Fig. 4:**
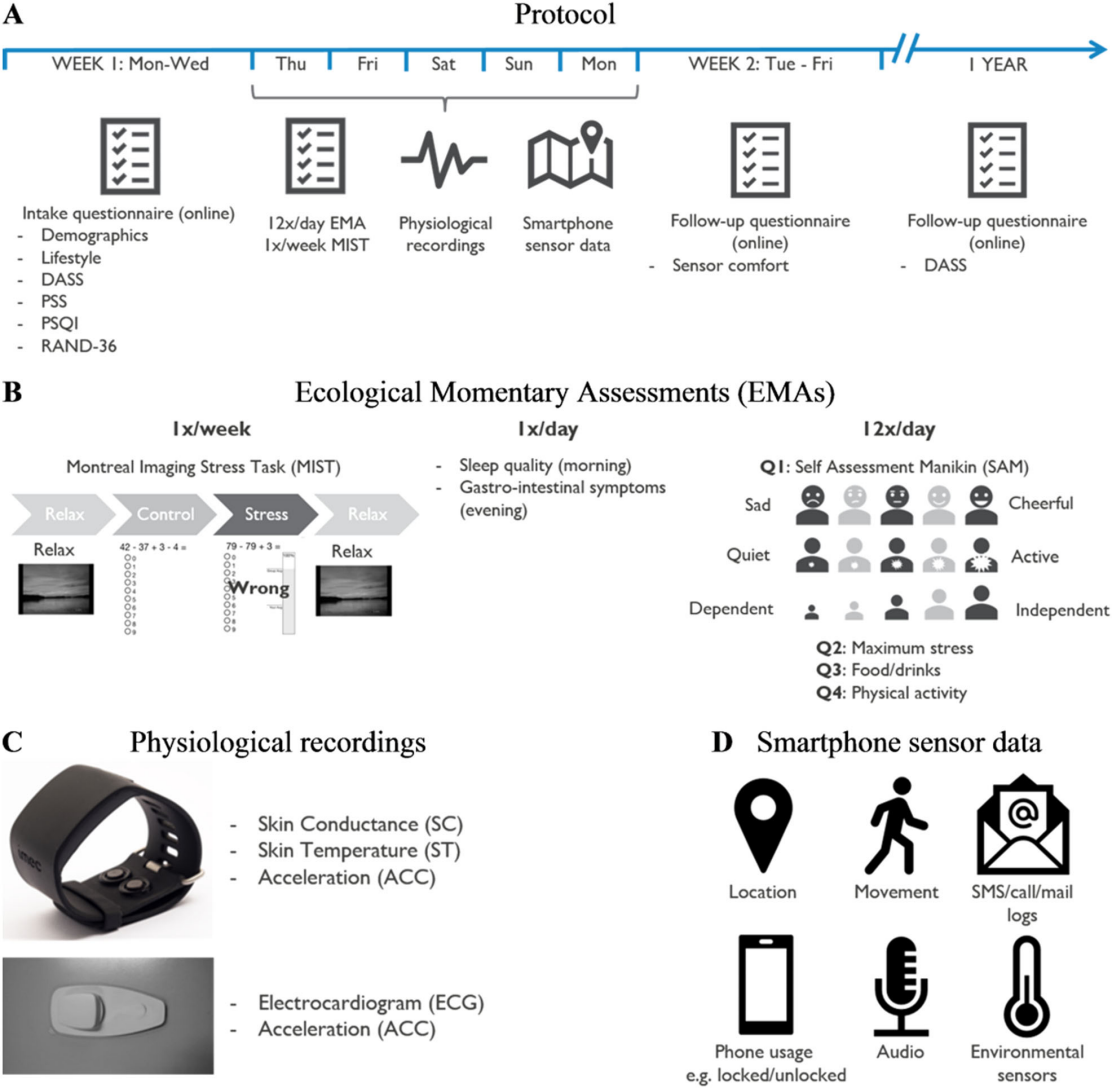
Study protocol. **a** Protocol timeline: starting with online intake questionnaires, followed by a 5-day trial, ending with a follow-up questionnaire just after the experiment and 1 year later. **b** Ecological momentary assessments (EMAs): once per subject the Montreal Imaging Stress Task is performed containing a series of mental arithmetic challenges. Once per day a sleep diary and gastro-intestinal symptoms diary are filled in and 12 times a day stress levels are recorded. **c** Physiological recordings: Chillband and chest patch to measure SC, ST, ECG, and acceleration. **d** Smartphone sensor data: overview of the data recorded

This observational study was approved by the Research Ethical Committee of UZ Leuven. The trial was conducted with 1002 subjects (484 male, 451 female, 67 did not fill in the questionnaire correctly), aged 39.4 ± 9.8, recruited in 11 technology-oriented, banking, and public sector companies. Subjects were included if they were active employees at the time of the study, no other inclusion or exclusion criteria were applied. Subjects did not receive any compensation for participating in the study apart from having a chance at winning a restaurant or travel voucher. The experiment was conducted over a time span of 2 years. The measurements lasted 5 days per subject, starting on Thursday morning and ending on Monday evening (Fig. 4). All subjects signed the informed consent before participating in the study.

Before the start of the experiment subjects completed an intake questionnaire (S1 Table). The first part inquired personal information such as age, gender, health problems, work situation, and lifestyle. Thereafter, four psychological questionnaires were used to assess baseline stress, depression, anxiety, sleep, and general health levels. These were the PSS,^6^ the PSQI,^27^ the DASS,^28^ and the RAND-36.^29^

On Thursday morning, the subjects received two wearable devices, along with a user manual and a USB-stick containing instructions on how to apply them. Two wearables were used to capture three physiological signals unobtrusively: the ECG, SC, and ST. Both the devices also measure 3D ACC, which signal was used for estimating intensity of physical activity and control for movement artifacts. Although EMG and BP are also frequently used in laboratory settings, these are less feasible to measure continuously in daily life. The first wearable was a chest patch (Fig. 4c), which received regulatory approval and is able to measure the ECG and ACC at a sampling rate of 256 and 32 Hz respectively. The second wearable was the imec’s Chillband (Fig. 4c), a wrist-worn device, designed to measure and record SC, ST, and ACC, sampled at 256, 1, and 32 Hz, respectively. Subjects were advised to wear the Chillband the entire day, but could take it off during the night, and to wear the chest patch the entire day and night. Subjects were asked to remove the Chillband while taking a shower and to remove Chillband and chest patch during vigorous physical activities. The battery life of both sensors exceeded the duration of the experiment. Data were recorded and stored on the devices’ internal SD cards and uploaded to a central data platform at the end of the experiment.

A custom-made smartphone application was used to trigger subjects to fill-out the EMAs (Fig. 4b). Previous research has shown correlations between stress and sleep efficiency^30^ and between stress and digestive diseases (e.g., irritable bowel syndrome).^31^ Therefore, the sleep quality of the previous night was inquired every morning and gastro-intestinal symptoms experienced during the day were inquired every evening with the Leuven Postprandial Distress Scale.^32^ Twelve times per day, the smartphone application requested the subjects to indicate their level of stress. The requests were sent at random times and at least 30 min apart. The request consisted of four brief questions (Q1–Q4 in Fig. 4b) pertaining to the past hour: first, the SAM^33^ was used as a visual scale to assess pleasure, arousal and dominance (i.e., level of control), i.e., affective emotions related to stress. The pleasure level could be used to differentiate “good” stress from “bad” stress, i.e., eustress versus distress, where eustress reflects the transition of the body to a lower allostatic load (i.e., “the price the body pays for being forced to adapt to unfavorable psychosocial or physical situations”^34^) and distress to a higher allostatic load.^34^ Second, the maximum stress level was annotated on a 5-point Likert scale, i.e., not at all, slightly, moderately, very and extremely stressed. Since eating and drinking behavior and physical activity can influence physiology,^13,35^ the third and fourth questions were used to indicate food and beverage consumption (i.e., caffeine, alcohol, soft drinks, breakfast, lunch, dinner, snack, or none) and activity levels (i.e., lying down, sitting, standing, walking, running, biking, driving the car, or something else), for which subjects could select multiple answers.

To assess individual physiological stress responses to a known common stressor, the Montreal Imaging Stress Task,^36^—based on the well-known Trier Social Stress Test—was included in the smartphone application (Fig. 4b). Each subject underwent this stress test during the first day of the experiment (Thursday) at a suitable moment (i.e., given enough time and in a quiet environment). The test consists of a 5 min rest period (relaxing music and images), a 5 min control period (simple mathematic tasks, no time restrictions or social control), a 5 min stress task (mathematic tasks with time restrictions and social control) and again a 5 min rest period (relaxing music and images).

Further, the smartphone application was used, conditional on subjects permission, to collect contextual data, i.e., location, smartphone usage, audio-features, movement, SMS/call/mail logs, and environmental sensors (Fig. 4d). Location data can be used to investigate the correlation between stress levels and locations, e.g., stress at home versus at work. Many research has already indicated that social support has a large influence on stress and health-related effects caused by stress.^37^ Audio features could be used to detect conversations and social interaction. SMS, call, and mail logs could be used as a proxy for workload and environmental sensors could provide a link between physiology and environment, e.g., higher environmental temperatures could be correlated with higher ST and SC.^38^

On Monday evening subjects returned the sensors. Finally, subjects completed a questionnaire about sensor comfort. As a follow-up on mental health, a year later a reminder was sent to retake the DASS.

### Data analysis—preprocessing

Raw sensor data and subject self-assessments were synchronized using UTC timestamps. Quality indicators and feature extraction algorithms were applied subsequently. Assessing the quality of the signals is necessary since these are prone to artifacts due to motion or incorrect sensor attachment.

The ECG quality indicator is based on Orphanidou et al.,^39^ which has shown a sensitivity for artifact detection of 94% and a specificity of 97%, and consists out of three rules and a template matching, verified on 10-s segments of ECG data: first, the extracted HR should be within 40 and 180 bpm. Second, the maximum gap between successive R-peaks cannot exceed 3 s. Third, the ratio of the maximum beat-to-beat interval to the minimum beat-to-beat interval within the segment should be less than 2.2. If all rules are satisfied, an adaptive QRS template matching is performed. The 10-s segment is either classified as of good or of bad quality.

In the SC quality indicator,^40^ the ratio of lost versus overall signal is calculated for each 5-s window. The signal is deemed lost if its value is below 0.001 µS. If this ratio is above 0.9, the signal is classified as of bad quality. Next, the algorithm searches for anomalies. For each second the maximum increase of a signal value is set to 20% and the maximum decrease to 10%, as suggested by Boucsein et al.^41^ If SC values within the segment do not satisfy these conditions the segment is classified as of bad quality.

Previous research defined the ST range at the wrist between 20 and 40 °C.^42^ Therefore, ST values outside this range are classified as of bad quality.

### Data analysis—physiological feature extraction

Eighteen physiological features of interested as investigated previously in refs ^10,11,23,43–49^ were included in our study: 6 features for ECG, including mean HR and time and frequency domain HRV features, 8 SC features, including tonic and phasic features, and 4 ST features. For accelerometer-based activity we included the standard deviation of the accelerometer magnitude (ACC SD).^16^ A complete list of all features is available in S5 Table, a codebook including the Python code to compute the features is included in S3 Text. All features were calculated in a window of 5 min with 4 min overlap. This is the minimum window required to calculate HRV features such as the root mean squared difference of successive RR intervals (RMSSD)^50^ due to the inherent regulation periodicity.^51^ The 4 min overlap was set to obtain a resolution of smoothed processed data of one sample per minute.

### Statistical analysis

Statistical tests were performed using the nonparametric Wilcoxon ranksum test for comparisons of continuous variables. To assess differences of continuous variables across multiple demographic groups we used the Kruskal–Wallis test. The *X*^2^ test was used for comparisons of categorical variables. Two-sided *p*-values of <0.05 were considered statistically significant. All statistical tests requiring multiple comparisons were corrected based on the Benjamini–Hochberg procedure with a false discovery rate of 0.05. Associations between longitudinal data (e.g., questionnaires presented 12 times per day, or continuous wearable data) were assessed using linear mixed effects models, using the lme4 R package,^52^ with self-reported pleasure or continuous wearable feature data as fixed effects and the subjects as random effect. A Gaussian family was used to model continuous variables (e.g., ACC SD), while a Poisson family was used to model stress responses. An ANOVA test was used to assess whether model parameters differed significantly from zero by comparing the change in model performance (Akaike’s information criterion) when a fixed effect (e.g., pleasure) was excluded from the model. Correlations between stationary data (e.g., questionnaires with single responses) were calculated using the Spearman correlation coefficient (*r*).

Location data were anonymized based on a random translation and rotation. Locations were clustered as unique stay locations, i.e., average location in more than 60 min within a radius of 1 km and commuting.

Only good quality physiological data (good QI in ≥80% of data points in the 5 min window) were used and features were normalized (*z*-normalization) per subject. Redundant features were removed based on correlations (max *r* = 0.7), resulting in a reduced feature set. Since self-reported stress responses (based on the maximum stress during the last hour, i.e., Q2 in Fig. 4b) were highly imbalanced (S2 Table), the three highest stress levels were merged, representing 14.3% of the data, so that three, instead of five, levels of stress (S1 = no stress, S2 = light stress, S3 = high stress) were considered.

Based on these data, associations between physiological features and self-reported stress levels were investigated. For each stress level the median of the normalized features across the entire population was calculated. Additionally, the median during the night (N) (00–06 am) was included as baseline. For each feature, the differences between medians of different states were computed: N–S1, N–S2, N–S3, S1–S2, S2–S3, and S1–S3. A Wilcoxon-test was performed to investigate significant differences and corrected for multiple comparisons.

A machine learning model was developed to predict stress levels based on physiological responses. Subjects reporting only one stress level (e.g., only “no stress”) were discarded. Since self-reported stress levels reflect the situation of the last hour, the stress value reported was registered for the 60 data points pertaining to that entire hour. We included only data for windows of at least 10 min of good quality and low physical activity (ACC SD ≤ 0.04, based on ref. ^16^ and adapted according to subject’s self-reported activity levels). A false discovery rate supervised feature selection was applied on the training set on uncorrelated features, according to the Benjamini–Hochberg procedure (Python scikit-learn, alpha = 0.05). We trained Random Forest models in a leave-one-subject-out approach. This means a model was trained based on the data of all subjects but one and tested on the data of that subject. This procedure was repeated until all subjects were tested exactly once. We used the F1-score, a weighted average between precision and recall, to evaluate the model’s performance on the left-out-subject. As comparison, we also calculated the F1-score for all subjects if the Random Forest model classified all samples as the majority class, i.e., S1.

We further evaluated subject’s physiological response, demographics, and psychological information based on individual model performance. Subjects were categorized in groups of low performance, with F1-score < 0.33 (performance as good as random), medium performance, with 0.33 < F1-score < 0.66 and high performance, with F1-score > 0.66. For each group we evaluated three characteristics: first, we evaluated the imbalance of the self-reported stress levels, as a higher imbalance (e.g., mainly reporting S1), could lead to a higher classification performance. Second, we investigated the average dynamic range of each group, where the dynamic range represents the average difference per physiological feature of each group between low (S1) and high (S3) self-reported stress levels. A higher dynamic range could be beneficial for model performance, as the feature can better differentiate between low and high stress. Third, we investigated subject’s demographics and psychological information based on the intake questionnaire. A Wilcoxon ranksum test was performed to investigate significant differences across low and high performance groups, we corrected for multiple comparisons. All data analyses were performed using Python (version 2.7).

### Code availability

All analyses were performed using Python (version 2.7) and scikit-learn (version 0.18.1). Detailed information on the functions that were used are listed in the Methods section. A detailed codebook on feature calculation is presented in S3 Text. Other code can be shared upon request to the authors.

## Electronic supplementary material

Supplemental material

## Data Availability

The data that support the findings of this study are available on request to the corresponding author. The data are not publicly available due to them containing information that could compromise research subject privacy.

## Supplementary material

**Supplementary information** accompanies the paper on the *npj Digital Medicine* website (10.1038/s41746-018-0074-9).

## References

[1] Selye H (1950). Stress and the general adaptation syndrome. Br. Med. J..

[2] Cohen S, Janicki-deverts D, Miller GE (2015). Psychological stress and disease. JAMA.

[3] Dimsdale JE (2008). Psychological stress and cardiovascular disease. J. Am. Coll. Cardiol..

[4] Khoury MJ, Iademarco MF, Riley WT (2016). Precision public health for the era of precision medicine. Am. J. Prev. Med..

[5] Jain SH, Powers BW, Hawkins JB, Brownstein JS (2015). The digital phenotype. Nat. Biotechnol..

[6] Lee EH (2012). Review of the psychometric evidence of the perceived stress scale. Asian Nurs. Res. (Korean Soc. Nurs. Sci.)..

[7] Alberdi A, Aztiria A, Basarab A (2016). Towards an automatic early stress recognition system for office environments based on multimodal measurements: a review. J. Biomed. Inform..

[8] Lovallo, W. R. *Stress & Health: Biological and Psychological Interactions*. SAGE Publications: California, US (2016).

[9] Sharma N, Gedeon T (2012). Objective measures, sensors and computational techniques for stress recognition and classification: a survey. Comput. Methods Prog. Biomed..

[10] Healey JA, Picard RW (2005). Detecting stress during real-world dring tasks using physiological sensors. IEEE Trans. Intell. Transp. Syst..

[11] Muaremi A, Arnrich B, Tröster G (2013). Towards measuring stress with smartphones and wearable devices during workday and sleep. Bionanoscience.

[12] Hovsepian, K. et al. cStress: towards a gold standard for continuous stress assessment in the mobile environment. in *Proceedings of the 2015 ACM International Joint Conference on Pervasive and Ubiquitous Computing—**UbiComp '15* 493–504 (2015). 10.1145/2750858.2807526 PMC4631393 26543926

[13] Sun, F.-T. et al. Activity-aware mental stress detection using physiological sensors. in *Proc. International Conference on Mobile Computing, Applications, and Services (MobiCASE)*, Vol. 76, 1–20 (2010).

[14] Smets E (2016). Comparison of machine learning techniques for psychophysiological stress detection. Comp. Mach. Learn. Tech..

[15] Sun FFT (2012). Activity-aware mental stress detection using physiological sensors. Mob. Comput..

[16] Rahman, M. M. et al. Are we there yet? Feasibility of continuous stress assessment via wireless physiological sensors. in *Proceedings of the 5th ACM Conference on Bioinformatics, Computational Biology, and Health Informatics—BCB’14*, 479–488 (2014). 10.1145/2649387.2649433 PMC4374173 25821861

[17] Wang, R. et al. Studentlife: assessing mental health, academic performance and behavioral trends of college students using smartphones. in *Proceedings of the 2014 ACM International Joint Conference on Pervasive and Ubiquitous Computing* 3–14 (2014).

[18] Finke JB, Kalinowski GI, Larra MF, Schächinger H (2018). The socially evaluated handgrip test: Introduction of a novel, time-efficient stress protocol. Psychoneuroendocrinology.

[19] Kirschbaum C, Pirke KM, Hellhammer DH (1993). The ‘Trier Social Stress Test’—a tool for investigating psychobiological stress responses in a laboratory setting. Neuropsychobiology.

[20] Billman GE (2013). The LF/HF ratio does not accurately measure cardiac sympatho-vagal balance. Front. Physiol..

[21] McNames J, Aboy M (2006). Reliability and accuracy of heart rate variability metrics versus ECG segment duration. Med. Biol. Eng. Comput..

[22] Healey, J. A. *Wearable and Automotive Systems for Affect Recognition from Physiology*. Ph.D. Thesis 158 (2000).

[23] Singh R, Conjeti S, Banerjee R (2013). A comparative evaluation of neural network classifiers for stress level analysis of automotive drivers using physiological signals. Biomed. Signal Process..

[24] Greco, A., Valenza, G. & Scilingo, E. P. *Advances in Electrodermal Activity Processing with Applications for Mental Health*. Springer International Publishing: New York, US (2016).

[25] Kistler A, Mariauzouls C, Von Berlepsch K (1998). Fingertip temperature as an indicator for sympathetic responses. Int. J. Psychophysiol..

[26] Carroll D, Ginty AT, Whittaker AC, Lovallo WR, de Rooij SR (2017). The behavioural, cognitive, and neural corollaries of blunted cardiovascular and cortisol reactions to acute psychological stress. Neurosci. Biobehav. Rev..

[27] Buysse DJ (1991). Quantification of subjective sleep quality in healthy elderly men and women using the Pittsburgh Sleep Quality Index (PSQI). Sleep.

[28] Henry JD, Crawford JR (2005). The short-form version of the Depression Anxiety Stress Scales (DASS-21): construct validity and normative data in a large non-clinical sample. Br. J. Clin. Psychol..

[29] Hays RD, Morales LS (2001). The RAND-36 measure of health-related quality of life. Ann. Med..

[30] Petersen H, Kecklund G, D’Onofrio P, Nilsson J, Åkerstedt T (2013). Stress vulnerability and the effects of moderate daily stress on sleep polysomnography and subjective sleepiness. J. Sleep. Res..

[31] Lee SP (2015). The effect of emotional stress and depression on the prevalence of digestive diseases. J. Neurogastroenterol. Motil..

[32] Carbone F (2016). Validation of the Leuven Postprandial Distress Scale, a questionnaire for symptom assessment in the functional dyspepsia/postprandial distress syndrome. Aliment. Pharmacol. Ther..

[33] Morris JD (1995). Observations: SAM: the self-assessment manikin: an efficient cross-cultural measurement of emotional response. J. Advert. Res..

[34] Kupriyanov RV, Sholokhov MA, Kupriyanov R, Zhdanov R (2014). The eustress concept: problems and outlooks. World J. Med. Sci..

[35] Acharya UR, Joseph KP, Kannathal N, Lim CM, Suri JS (2006). Heart rate variability: a review. Med. Biol. Eng. Comput..

[36] Dedovic K, Renwick R, Mahani NK, Engert V (2005). The Montreal Imaging Stress Task: using functional imaging to investigate the effects of perceiving and processing psychosocial stress in the human brain. Psychiatry Neurosci..

[37] Van der Doef M, Maes S (1999). The Job Demand-Control (-Support) Model and psychological well-being: a review of 20 years of empirical research. Work Stress.

[38] Vetrugno R, Liguori R, Cortelli P, Montagna P (2003). Sympathetic skin response. Clin. Auton. Res..

[39] Orphanidou, C. et al. Signal quality indices for the electrocardiogram and photoplethysmogram: derivation and applications to wireless monitoring. *IEEE J. Biomed. Health Inform.* **19**, 832–838 (2014). 10.1109/JBHI.2014.2338351 25069129

[40] Kocielnik, R., Sidorova, N., Maggi, F. M., Ouwerkerk, M. & Westerink, J. H. D. M. Smart technologies for long-term stress monitoring at work. in *Proceedings of the 26th IEEE International Symposium on Computer-Based Medical Systems* 53–58 (IEEE, 2013).

[41] Boucsein W (2012). Publication recommendations for electrodermal measurements. Psychophysiology.

[42] Jones, L. A. & Lederman, S. J. *Human Hand Function*. Oxford University Press: Oxford, UK (2006).

[43] Han L (2017). Detecting work-related stress with a wearable device. Comput. Ind..

[44] Xu Q, Nwe TL, Guan C (2015). Cluster-based analysis for personalized stress evaluation using physiological signals. IEEE J. Biomed. Health Inform..

[45] Zhai, J. & Barreto, A. Stress detection in computer users based on digital signal processing of noninvasive physiological variables. in *Proc. Annual International Conference of the IEEE Engineering in Medicine and Biology Society**, EMBS* 1355–1358 (2006). 10.1109/IEMBS.2006.259421 17946041

[46] de Vries GJJ, Pauws SC, Biehl M (2015). Insightful stress detection from physiology modalities using Learning Vector Quantization. Neurocomputing.

[47] Karthikeyan P, Murugappan M, Yaacob S (2013). Multiple physiological signal-based human stress identification using non-linear classifiers. Elektron. Elektrotech..

[48] Sano, A. & Picard, R. W. Stress recognition using wearable sensors and mobile phones. in *Proc.**Humaine Association Conference on Affective Computing and Intelligent Interaction* 671–676 (2013).

[49] Wijsman, J., Grundlehner, B., Liu, H., Penders, J. & Hermens, H. Wearable physiological sensors reflect mental stress state in office-like situations. in *Proc. Humaine Association Conference on Affective Computing and Intelligent Interaction, ACII 2013* 600–605 (2013).

[50] Guidelines: Heart rate variability standards of measurement, physiological interpretation, and clinical use. *Eur. Heart J*. **17**, 354–381 (1996). 8737210

[51] Grant CC, van Rensburg DCJ, Strydom N, Viljoen M (2011). Importance of tachogram length and period of recording during noninvasive investigation of the autonomic nervous system. Ann. Noninvasive Electrocardiol..

[52] Bates D, Mächler M, Bolker B, Walker S (2015). Fitting linear mixed-effects models using lme4. J. Stat. Softw..

[53] Symonds MRE, Moussalli A (2011). A brief guide to model selection, multimodel inference and model averaging in behavioural ecology using Akaike's information criterion. Behav. Ecol. Sociobiol..

[54] Steptoe A, Butler N (1996). Sports participation and emotional wellbeing in adolescents. Lancet.

[55] Stubbs B (2017). Perceived stress and smoking across 41 countries: a global perspective across Europe, Africa, Asia and the Americas. Sci. Rep..

[56] Rius C, Fernandez E, Schiaffino A, Borràs JM, Rodríguez-Artalejo F (2004). Self perceived health and smoking in adolescents. J. Epidemiol. Community Health.

[57] Watson EJ, Coates AM, Kohler M, Banks S (2016). Caffeine consumption and sleep quality in Australian adults. Nutrients.

[58] Smagula SF, Stone KL, Fabio A, Cauley JA (2016). Risk factors for sleep disturbances in older adults: evidence from prospective studies. Sleep Med. Rev..

[59] Irish LA, Kline CE, Gunn HE, Buysse DJ, Hall MH (2015). The role of sleep hygiene in promoting public health: a review of empirical evidence. Sleep Med. Rev..

[60] Stewart, A. & Ware, J. *Measuring Functioning and Well-Being: The Medical Outcomes Study Approach*. Duke University Press: Durham, North Carolina, US (1992).

