# Supplementary material for: Large-scale wearable data reveal digital phenotypes for daily-life stress detection
Source: NPJ Digit Med. 2018 Dec 12;1:67. doi: 10.1038/s41746-018-0074-9 (PMC6550211; doi:10.1038/s41746-018-0074-9)
Supplement: Supplementary file 1 — Supplemental material [file 41746_2018_74_MOESM1_ESM.docx]

Supplementary Information

**The supplementary information includes**

Text S1 to S3

Figs S1 to S2

Tables S1 to S6

**S1 Text. Associations between questionnaire-based lifestyle and health indicators.**

Data on lifestyle, general health and health indicators (validated psychological questionnaires, i.e. PSQI, DASS, PSS and RAND-36) were collected at the start of the experiment and are available for 932 subjects. Associations between several aspects of the subject's lifestyle (sports, diet and habits) and health indicators (RAND-36, PSS and PSQI) are shown in S1 Fig. RAND-36 taps eight health concepts, ranging from physical functioning to emotional well-being and social functioning perceptions ^29^. A positive correlation was found between energy levels and emotional wellbeing (*r*=0.66), with increased levels of energy levels and emotional wellbeing for subjects who practice sports more often (>5 weekly sports hours; emotional well-being: 78.0±14.0; energy: 69.2±16.3) compared to subjects who do not exercise often (0-1 weekly sports hours; emotional well-being: 70.0±14.5; energy: 56.9±17.8; kruskal-wallis *p* = 0.004, S1A Fig). This positive relation between sports and emotional wellbeing has long been recognized and is here confirmed ^54^. Furthermore, self-perceived stress (based on PSS) was negatively correlated (r = -0.75) with emotional wellbeing. PSS was higher for subjects who reported medication intake (15.2±6.4), compared to subjects with no medication (14.0±5.9, ranksums *p* = 0.013, S1B Fig). Subjects who smoke tended to report higher levels of self-perceived stress (PSS), and tended to rate their lifestyle as being less healthy (RAND36 General Health: 35.0±7.1) than those who do not smoke (RAND36 General Health: 68.4±16.3; kruskal-wallis *p* = 0.07, S1C Fig). These results are in line with earlier reports indicating that a one-unit increase in PSS results in a 5% increased odds of smoking ^55^, and that people who smoke rate their lifestyle as less healthy than people who do not ^56^.

We found no correlation between BMI and general health (r = -0.12); however, we observed decreasing general health values with increasing number of take-out food times through the week (No take-out: 70.4±15.9 vs ≥5 times: 62.5±18.1; kruskal-wallis p = 0.008; S1D Fig). There was no correlation between caffeinated beverages consumption and PSQI scores (PSQI scores higher than 5 indicate worse sleep quality; S1E Fig), although literature indicates that people with lower sleep quality on average consume more caffeine ^57^. Women tend to have inferior sleep quality than men (5.2±2.8 vs 4.7±2.4, respectively; ranksums *p* = 0.023), as is confirmed by literature ^58^. We also found a negative correlation between energy levels and PSQI, indicating that subjects with inferior sleep quality have lower energy (r=-0.47). There was no significant difference on PSQI with alcohol intake (ranksums *p* = 0.13; S1F Fig). Literature suggests that for non-dependent alcohol users (e.g. light/occasional, habitual weekend use), alcohol consumption just before bedtime can reduce sleep quality ^59^. However, the impact of afternoon or early evening alcohol consumption on sleep quality is not yet well understood ^59^.

All together these findings represent a large-scale verification supporting previous work and confirm the value of data sampled with validated questionnaires: lifestyle and health indicators are strongly linked, underlining the need for behavior change interventions that support preventive healthcare.

**S2 Text. Comparison of feature dynamic range in low and high classification performance groups.**

Subjects were subdivided based on their classification performance (i.e. F1-score) into low, medium and high performance groups. In the main text the differences in dynamic range were shown (i.e. the average difference on physiological features between periods with low (S1) and high (S3) self-reported stress levels) between these performance groups, for ECG mean HR, SC phasic and ST median. In S2 Fig these differences were shown for the remaining features. For 15 out of 18 features the dynamic range of the high performance group was larger than that of the low performance group, only for ECG SDNN, ECG LFHF and ST SD this was the opposite. Additionally, for 10 out of 18 features (ECG LF, ECG HF, SC mean, SC phasic, SC RR, SC diff2, SC R, SC dur, ST mean, ST median) the dynamic range of the high performance group was larger than that of the medium performance group *and* the dynamic range of the medium performance group was larger than that of the low performance group, meaning the dynamic range decreased monotonically from high to low performance group.

To assess if this trend could be due to chance, we subdivided subjects randomly, instead of based on their F1-score, and investigated the dynamic range for each group. For each feature we calculated the dynamic range per group (i.e. absolute value of (mean(high_stress) – mean(low_stress))). First, only two groups were compared and the number of features for which the dynamic range of one group was larger than for the other group was calculated. Then, all three groups were compared and the number of features for which there was a monotonic increase/decrease in dynamic range across the three groups was calculated. This analysis was repeated 100 times.

Based on a random division of subjects into two groups, on average 12 out of 18 features were found for which the dynamic range of one group was larger than the other group. Based on 100 repetitions, the 95% confidence interval of number of features for which the dynamic range of one group was larger than the other group was [11-16]. When dividing subjects based on their F1-score, 15 features were found for which the dynamic range of the high performance group was larger than of the low performance group. This falls within the 95% confidence interval, suggesting that this result could be found by chance. However, we also found that on average 5 out of 18 features showed a monotonic increase/decrease in dynamic range with a 95% confidence interval of [2-9] features. When dividing subjects based on their F1-score, 10 features were found for which there was a monotonic decrease in dynamic range across the three groups (from high to low performance groups), indicating that this finding was unlikely to be due to chance.

**S3 Text. Codebook physiological feature calculation (Python version 2.7)**

"""

Codebook for physiological feature calculation for stress detection

input:

- Array of data in 5 min window

- Heart rate (HR)

- Skin conductance (SC)

- Skin temperature (ST)

output:

- Feature output values for HR, SC and ST

"""

**import** numpy **as** np

**import** pandas **as** pd

**from** scipy **import** signal

""" ECG FEATURES """

## HR represents the array of HRs as calculated in the 5 minute window

## beat_interval represents the array of beat intervals in the 5 minute window

## ECG mean HR

ECG_mean_HR**=**np**.**mean**(**HR**)**

## ECG SDNN

ECG_SDNN**=**np**.**std**(**beat_interval**)**

## ECG RMSSD

ECG_RMSSD**=**np**.**sqrt**(**np**.**mean**(**beat_interval******2**))**

## ECG LF / ECG HF / ECG LFHF

# beat_interval_interp represents the 4Hz interpolated beat interval

signal_length **=** len**(**beat_interval_interp**)**

hann_window **=** np**.**hanning**(**signal_length**)**

hann_window **=** hann_window**/**np**.**sum**(**hann_window**)**

windowed_signal **=** beat_interval_interp*****hann_window

windowed_signal_fft **=** np**.**abs**(**np**.**fft**.**fft**(**windowed_signal**))** #Fast fourier

windowed_signal_power **=** windowed_signal_fft******2

LF_start **=** np**.**round**(**0.05**/(**4**/**signal_length**))**

LF_stop **=** np**.**round**(**0.15**/(**4**/**signal_length**))**

HF_start **=** np**.**round**(**0.15**/(**4**/**signal_length**))**

HF_stop **=** np**.**round**(**0.4**/(**4**/**signal_length**))**

ECG_LF **=** np**.**sum**(**windowed_signal_power**[**int**(**LF_start**):**int**((**LF_stop**))])**

ECG_HF **=** np**.**sum**(**windowed_signal_power**[**int**(**HF_start**):**int**((**HF_stop**))])**

ECG_LFHF **=** ECG_LF/ECG_HF

""" SC FEATURES """

## SC represents the array of SC values in the 5 minute window as a

#(pandas dataframe)

## SC mean

SC_mean**=**np**.**mean**(**SC**)**

## SC phasic

SC_phasic **=** **(**SC******2**).**sum**()** **/** SC**.**shape**[**0**]**

##SC RR / SC_R

fs**=**256 #Sampling frequency

smooth_length**=**3 #smooth at 3 seconds

sc **=** pd**.**rolling_mean**(**SC**,** int**(**smooth_length ***** fs**))** # smooth the signal

scdiff **=** pd**.**rolling_mean**(**sc**.**diff**(),** int**(**fs**))** ***** fs # Calculate derivative and

# smooth at 1 s

threshold**=**0.0093 #empirically defined threshold

scdiff**[**scdiff **<** threshold**]** **=** 0

scdiff**.**iloc**[**0**:**int**(**fs**/**2**)]** **=** 0**;** # Set first half second to zero to omit

#boundary effects of the smoothing operation

scdiff**.**iloc**[-**int**(**fs**/**2**):]** **=** 0**;** # Set last half second to zero to omit

#boundary effects of the smoothing operation

a **=** np**.**nonzero**(**np**.**array**(**scdiff**))** # indices of samples above the threshold

b **=** np**.**diff**(**a**)**

SC_R **=** **[]**

lb **=** len**(**b**)**

**for** m **in** range**(**lb**):**

**if** **(**b**[**m**]** **>** 1**)** **or** **(**m **==** lb**):** # gap in sample indices above the threshold or

#last part of signal above threshold

i **=** a**[**m**]**

c **=** scdiff**[**i**]**

**while** **(**c **>** 0**)** **and** **(**i **<** len**(**scdiff**)):** # as long as the derivative is

#above zero and we're not at the end of the signal yet

i **=** i **+** 1 # go to the next sample

c **=** scdiff**.**iloc**[**i**]**

SC_R**.**append**(**i**)** # when the derivative becomes negative, add the

#peak to the scr vector

SC_RR **=** 1.0*****len**(**SC_R**)** **/** **(**len**(**SC**)** **/** fs**)**

## SC diff2

sc**=**SC**.**diff**(**2**)**

SC_diff2**=(**sc******2**).**sum**()** **/** sc**.**shape**[**0**]**

## SC mag / SC dur / SC area

#Filter out the high frequency noise

b**,**a**=**signal**.**ellip**(**4**,** 0.1**,** 40**,** 4*****2**/**float**(**fs**))**

sc_filtered**=**np**.**transpose**(**signal**.**filtfilt**(**b**,**a**,**np**.**transpose**(**SC**.**values**)))**

#Find significant startles

#Set a threshhold to define significant startle

thresh**=**0.01

vector**=**np**.**zeros**(**len**(**sc_filtered**))**

sfprime**=**np**.**zeros**(**len**(**sc_filtered**))**

**for** i **in** range**(**len**(**sc_filtered**)-**fs**):**

sfprime**[**i**+**fs**/**2**]=**sc_filtered**[**i**+**fs**-**1**]-**sc_filtered**[**i**]**

**if** sfprime**[**i**+**fs**/**2**]>**thresh**:**

vector**[**i**+**fs**/**2**]=**1

overthresh**=**np**.**nonzero**(**vector**)[**0**]** #overthresh is the values at which the segment

#is over the threshold

gaps**=**np**.**diff**(**overthresh**)**

big_gaps**=**np**.**where**(**gaps**>**fs**)[**0**]**

iend**=**np**.**array**([])**

ibegin**=**np**.**array**([])**

**for** i **in** range**(**len**(**big_gaps**)):**

iend**=**np**.**append**(**iend**,** overthresh**[**big_gaps**[**i**]])**

ibegin**=**np**.**append**(**ibegin**,** overthresh**[**big_gaps**[**i**]+**1**])**

#Fine tuning

#The idea is to find the zero crossing closest to where it goes over threshold

#Find all zero crossings

overzero**=**np**.**where**(**sfprime**>**0**)[**0**]**

zerogaps**=**np**.**diff**(**overzero**)**

z_gaps**=**np**.**where**(**zerogaps**>**1**)[**0**]**

iup**=[]**

idown**=[]**

**for** i **in** range**(**len**(**z_gaps**)):**

idown**=**np**.**append**(**idown**,** overzero**[**z_gaps**[**i**]])**

iup**=**np**.**append**(**iup**,** overzero**[**z_gaps**[**i**]+**1**])**

#find up crossing closest to ibegin

new_begin**=**np**.**zeros**(**len**(**ibegin**))**

**for** i **in** range**(**len**(**ibegin**)):**

temp**=**np**.**where**(**iup**<**ibegin**[**i**])[**0**]**

**if** temp**.**size**==**0**:**

**continue**

choice**=**temp**[-**1**]**

new_begin**[**i**]=**iup**[**choice**]**

#to find the end of the startle, find the maximum between startle beginnings

new_end**=**np**.**zeros**(**len**(**iend**))**

**for** i **in** range**(**len**(**new_begin**)-**1**):**

startit**=**new_begin**[**i**]**

endit**=**new_begin**[**i**+**1**]**

**if** **not** startit**==**endit**:**

loc**=**np**.**argmax**(**s**[**int**(**startit**):**int**(**endit**)])**

new_end**[**i**]=**startit**+**loc

**else:**

new_end**[**i**]=**startit

**if** len**(**new_begin**)>**0**:**

lastbegin**=**new_begin**[**len**(**new_begin**)-**1**]**

lastloc**=**np**.**argmax**(**SC**.**values**[**int**(**lastbegin**):**len**(**SC**.**values**)-**1**])**

new_end**[**len**(**new_begin**)-**1**]=**new_begin**[**len**(**new_begin**)-**1**]+**lastloc

SC_mag**=**np**.**zeros**(**len**(**new_end**))** #initialize a vector of startle magnitudes

SC_dur**=**np**.**zeros**(**len**(**new_end**))** #initialize a vector of startle durations

**for** i **in** range**(**len**(**new_end**)):**

SC_dur**[**i**]=**new_end**[**i**]-**new_begin**[**i**]**

SC_mag**[**i**]=**SC**.**values**[**new_end**[**i**]]-**SC**.**values**[**new_begin**[**i**]]**

SC_area**=**np**.**multiply**(**SC_dur**,** SC_mag**)/**2

SC_dur**=**np**.**sum**(**SC_dur**)**

SC_mag**=**np**.**sum**(**SC_mag**)**

SC_area**=**np**.**sum**(**SC_area**)**

""" ST FEATURES """

## ST represents the array of ST values in the 5 minute window as a

#(pandas dataframe)

#ST mean

ST_mean**=**ST**.**mean**()**

#ST median

ST_median**=**ST**.**median**()**

# ST SD

ST_SD**=**ST**.**std**()**

#ST slope

line_fit**=**np**.**polyfit**(**range**(**len**(**ST**.**values**)),**ST**.**values**,**1**)** #fit line through data

ST_slope**=**line_fit**[**0**]**

**
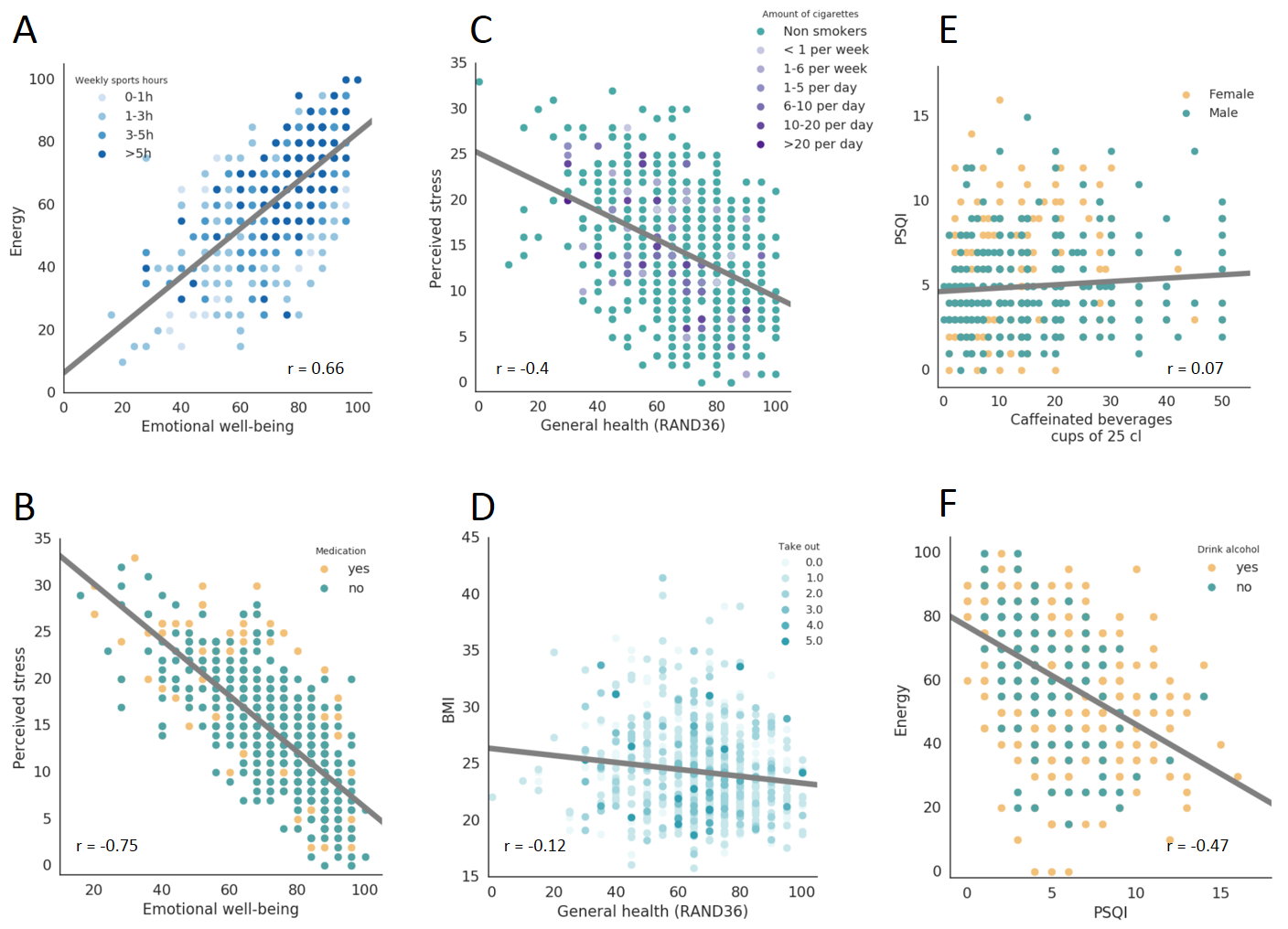
**

**S1 Fig. Associations between questionnaire-based lifestyle and health indicators.**

Detailed information is available in S1 Text. Health indicators based on psychological questionnaires: RAND-36, PSS and PSQI. A) Correlation between emotional wellbeing and energy reinforced by practicing sports. B) Correlation between perceived stress and emotional well-being, reinforced by medication intake. C) Negative correlation between perceived stress and general health, reinforced by smoking. D) No significant correlation between BMI and general health, but worse general health levels for people who eat more take-out food. E) No significant correlation between PSQI and caffeine consumption, women showed worse sleep quality. F) Negative correlation between energy and PSQI, not linked to alcohol consumption.


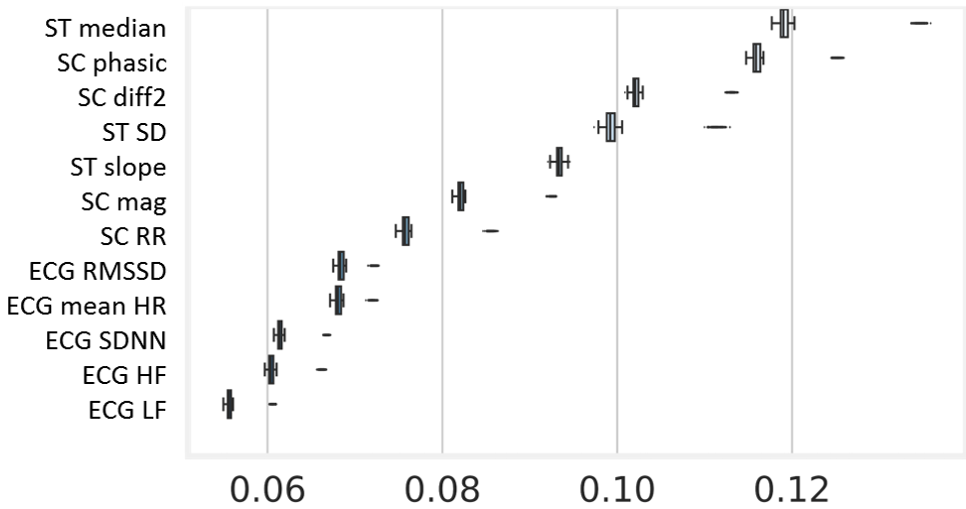


**S2 Fig. Boxplot of the feature importance across subjects.**

The x-axis represents the feature importance (range 0-1), the y-axis the features. The boxplot line represents the median, the box extends from the lower to upper quartile values, whiskers extend from minimum to maximum (indicating the range), and flier points are considered outliers.


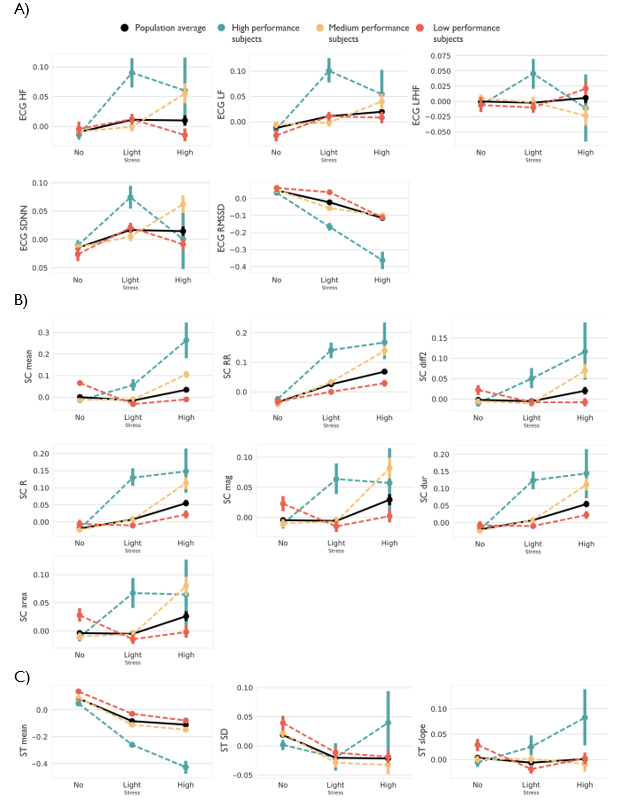


**S3 Fig. Comparison of dynamic ranges per feature across low, medium and high classification performance groups.**

In A, B and C average ECG, SC and ST related features are shown respectively for low (red), medium (yellow) and high performance (green) groups and compared with the entire population average in phases of no, light and high stress. The high performance group has a larger dynamic range, i.e. larger difference between physiology in no stress and high stress situations, for 16 out of 18 features as compared to the low performance group.

**S1 Table. Overview of intake questionnaire.**

For continuous variables the mean and standard deviation of the population (932 subjects who filled in the questionnaire) are presented, for categorical variables the percentage of the population for each class is presented. Subjects not answering the specific question are denoted by answer value ‘NaN’.

| **Variable** | **Classes** |
| --- | --- |
| Age | Mean = 39.5, SD = 9.8 |
| Length (cm) | Mean = 170.1, SD = 26.9 |
| Weight (kg) | Mean = 73.8, SD = 14.5 |
| Gender | Female (n=446, 44.5%) |
|  | Male (n=481, 48.0%) |
|  | NaN (n=75, 7.5%) |
| Origin | Africa (n=7, 0.7%) |
|  | Asia (n=45, 4.5%) |
|  | Europe (n=854, 85.2%) |
|  | South America (n=13, 1.3%) |
|  | Central America (n=3, 0.3%) |
|  | North America (n=6, 0.6%) |
|  | NaN (n=74, 7.4%) |
| Marital status | Single (n=183, 18.3%) |
|  | Cohabiting (n= 229, 22.9%) |
|  | Married (n=450, 44.9%) |
|  | Divorced (n=57, 5.7%) |
|  | Widowed (n=9, 0.9%) |
|  | NaN (n=74, 7.4%) |
| Children | Yes (n=549, 54.8%) |
|  | No (n= 380, 37.9%) |
|  | NaN (n=73, 7.3%) |
| If “Yes” to “Children”: how many | Mean = 2.1, SD = 0.8 |
| Pregnant | Yes (n=9, 0.9%) |
|  | No (n=919, 91.7%) |
|  | NaN (n=74, 7.4%) |
| Healthy lifestyle (1 = unhealthy – 10 = healthy) | Mean = 6.0, SD = 1.8 |
| Sports | Yes (n= 687, 68.6%) |
|  | No (n=251, 25.0%) |
|  | NaN (n=64, 6.4%) |
| If “Yes” to “Sports”: Hours of sports per week | 0-1h (n=118, 11.8%) |
|  | 1-3h (n=335, 33.4%) |
|  | 3-5h (n=153, 15.3%) |
|  | >5h (n=79, 7.9%) |
|  | NaN (n=317, 31.6%) |
| If “No” to “Sports”: other hobbies | Yes (n=182, 18.2%) |
|  | No (n=69, 6.9%) |
|  | NaN (n=751, 74.9%) |
| Smoke | Yes (n=74, 7.4%) |
|  | No (n=856, 85.4%) |
|  | NaN (72, 7.2%) |
| If “Yes” to “Smoke”: how many cigarettes | Less than 1 per week (n=6, 0.6%) |
|  | 1-6 per week (n=17, 1.7%) |
|  | 1-5 per day (n=16, 1.6%) |
|  | 6-10 per day (n=24, 2.4%) |
|  | 10-20 per day (n=12, 1.2%) |
|  | >20 per day (n=2, 0.2%) |
|  | NaN (n=925, 92.3%) |
| Caffeinated beverages | Yes (n=796, 79.4%) |
|  | No (n=132, 13.2%) |
|  | NaN (n=74, 7.4%) |
| If “Yes” to “caffeinated beverages”: how many cups per week | Mean = 15.5, SD = 10.8 |
| Alcohol | Yes (766, 76.4%) |
|  | No (n=163, 16.3%) |
|  | NaN (n=73, 7.3%) |
| If “Yes” to “Alcohol”: how many glasses per week | Mean = 6.9, SD = 6.9 |
| Fruit and vegetables | No (n=9, 0.9%) |
|  | 1-6 portions per week (n=156, 15.6%) |
|  | 1 portion per day (n=179, 17.9%) |
|  | 2-3 portions per day (n=410, 40.9%) |
|  | 4-5 portions per day (n=132, 13.2%) |
|  | >5 portions per day (n=39, 3.9%) |
|  | NaN (n=77, 7.7%) |
| Nr of take-out meals per week | Mean = 1.0, SD =1.0 |
| Diet | Pescetarian (n=22, 2.2%) |
|  | Vegetarian (n=24, 2.4%) |
|  | Vegan (n=7, 0.7%) |
|  | None of the above (n=867, 86.5%) |
|  | NaN (n=82, 8.2%) |
| Medication | Yes (n=305, 30.5%) |
|  | No (n=614, 61.3%) |
|  | NaN (n=83, 8.3%) |
| If “Yes” to “Medication”: Which medication | *Open text* |
| Current heart disease | Yes (n=26, 2.6%) |
|  | No (n= 893, 89.1%) |
|  | NaN (n=83, 8.3%) |
| If “Yes” to “Current heart disease”: Which disease | *Open text* |
| Heart disease in the past | Yes (n=23, 2.3%) |
|  | No (n= 894, 89.2%) |
|  | NaN (n=85, 8.5%) |
| If “Yes” to “Heart disease in the past”: Which disease | *Open text* |
| Chronic disease | Yes (n=113, 11.3%) |
|  | No (n=793, 79.1%) |
|  | NaN (n=96, 9.6%) |
| If “Yes” to “Chronic disease”: Which disease | *Open text* |
| Education | Primary school (n=2, 0.2%) |
|  | Secondary school (n=62, 6.2%) |
|  | Bachelors (n=303, 30.2%) |
|  | Masters/PhD (n=509, 50.8%) |
|  | NaN (n=126, 12.6%) |
| Employee type | Full-time (n=606, 60.5%) |
|  | Part-time (n=152, 15.2%) |
|  | Shift worker (n=16, 1.6%) |
|  | Interim (n=1, 0.1%) |
|  | Consultant (n=13, 1.3%) |
|  | PhD (n=38, 3.8%) |
|  | Intern/student (n=20, 2.0%) |
|  | NaN (n=156, 15.6%) |
| If “Employee type” is “part-time”: Percent work | Mean = 77.5, SD = 13.8 |
| People manager | Yes (n=159, 15.9%) |
|  | No (n=713, 71.2%) |
|  | NaN (n=130, 12.9%) |
| If “Yes” to “People manager”: how many persons | 1-5 (n= 27, 2.7%) |
|  | 6-10 (33, 3.3%) |
|  | 11-20 (n=31, 3.1%) |
|  | 21-50 (n=22, 2.2%) |
|  | 51-100 (n=6, 0.6%) |
|  | >100 (n=8, 0.8%) |
|  | NaN (n=873, 87.1%) |
| PSQI | Mean = 4.9, SD = 2.6 |
|  |  |
|  | Good sleep (<5) (n= 439, 43.8%) |
|  | Poor sleep (>=5) (n= 402, 40.1%) |
|  | NaN (n=161, 16.1%) |
| DASS - Depression | Mean = 2.6, SD = 3.1 |
|  |  |
|  | Normal (0-4) (n= 692, 69.1%) |
|  | Mild (5-6) (n= 80, 8.0%) |
|  | Moderate (7-10) (n= 75, 7.5%) |
|  | Severe (11-13) (n= 17, 1.7%) |
|  | Extremely severe (>=14) (n=7, 0.7%) |
|  | NaN (131, 13.1%) |
| DASS - Anxiety | Mean = 2.0, SD = 2.6 |
|  |  |
|  | Normal (0-3) (n= 706, 70.5%) |
|  | Mild (4-5) (n= 87, 8.7%) |
|  | Moderate (6-7) (n= 35, 3.5%) |
|  | Severe (8-9) (n=21, 2.1%) |
|  | Extremely severe (>=10) (n=20, 2.0%) |
|  | NaN (n=133, 13.3%) |
| DASS - Stress | Mean = 5.1, SD = 3.8 |
|  |  |
|  | Normal (0-7) (n=661, 66.0%) |
|  | Mild (8-9) (n=97, 9.7%) |
|  | Moderate (10-12) (n=70, 7.0%) |
|  | Severe (13-16) (n= 38, 3.8%) |
|  | Extremely severe (>=17) (n= 7, 0.7%) |
|  | NaN (n=129, 12.9%) |
| PSS | Mean = 14.4, SD = 6.1 |
|  |  |
|  | Very low (0-7) (n=121, 12.1%) |
|  | Low (8-11) (n=171, 17.1%) |
|  | Average (12-15) (n=224, 22.4%) |
|  | High (16-20) (n=213, 21.3%) |
|  | Very high (>=21) (n=146, 14.6%) |
|  | NaN (n=127, 12.7%) |
| RAND-36 - physical functioning | Mean = 89.6, SD = 15.8 |
|  | Results for baseline of the Medical Outcomes Study^60^ (n=2471): 70.6 ± 27.4 |
| RAND-36 - bodily pain | Mean = 85.7, SD = 16.1 |
|  | Results for baseline of the Medical Outcomes Study^60^ (n=2471): 70.8 ± 25.5 |
| RAND-36 - role limitations due to physical health problems | Mean = 85.2, SD = 28.9 |
|  | Results for baseline of the Medical Outcomes Study^60^ (n=2471): 53.0 ± 40.8 |
| RAND-36 - role limitations due to personal or emotional problems | Mean = 80.5, SD = 32.5 |
|  | Results for baseline of the Medical Outcomes Study (60) (n=2471): 65.8 ± 40.7 |
| RAND-36 - emotional well-being | Mean = 72.4, SD = 15.3 |
|  | Results for baseline of the Medical Outcomes Study^60^ (n=2471): 70.4 ± 22.0 |
| RAND-36 - social functioning | Mean = 84.6, SD = 19.0 |
|  | Results for baseline of the Medical Outcomes Study^60^ (n=2471): 78.8 ± 25.4 |
| RAND-36 - energy/fatigue | Mean = 60.5, SD = 19.2 |
|  | Results for baseline of the Medical Outcomes Study^60^ (n=2471): 52.2 ± 22.4 |
| RAND-36 - general health perceptions | Mean = 67.9, SD = 16.4 |
|  | Results for baseline of the Medical Outcomes Study^60^ (n=2471): 57.0 ± 21.11 |
|  |  |

**S2 Table. Overview of self-reported stress responses.**

| **Variable** | **Dataset information** |
| --- | --- |
| Nr of subjects with at least one report | 920 subjects |
| Total nr of responses | 23,429 stress reports |
| Average nr of responses per subject | Mean = 25, range: 1 – 54 |
| Average compliance per subject | Mean = 42%, range: 2%-90% |
| Division self-reported stress levels | 53.4% no stress, 32.3% light stress, 11.4% moderate stress, 2.6% high stress and 0.3% extremely high stress |
| Average nr of responses per subject on Thursday | Mean = 6 , SD = 2.68 |
| Average nr of responses per subject on Friday | Mean = 6.65 , SD = 2.87 |
| Average nr of responses per subject on Saturday | Mean = 6.17, SD = 2.81 |
| Average nr of responses per subject on Sunday | Mean = 6.20, SD = 2.86 |
| Average nr of responses per subject on Monday | Mean = 4.7, SD = 2.14, significantly lower than other days (Wilcoxon ranksum *p* < 0.001) |

**S3 Table. Overview of smartphone-based sensor data.**

One data point reflects one minute of information. The average coverage is calculated by dividing the number of data points (represented in timeframes of minutes) by the total time of the study in minutes. Location could be monitored when the self-reporting mobile app was open or it could be monitored continuously (i.e. every 15 minutes). Unique locations were calculated as the average location in more than 60 min within a radius of 1km. Audio features include amplitude and variance of the sound signal and likelihood of voice activity.

|  | Nr of subjects with at least one datapoint recorded | Average nr of data points per subject | Average coverage across 5 days of trial | Unique locations per subject |
| --- | --- | --- | --- | --- |
| Location recorded when app was open | 612 subjects | Mean = 35,  range: 1-72 | Mean = 0.6 % ,  range: 0.02-1.2% | 5 unique locations |
| Location recorded continuously | 312 subjects | Mean = 452,  range: 1-3292 | Mean = 7.2%,  range: 0.02-52.8% | 5 unique locations |
| Audio features | 240 subjects | Mean = 51,  range: 1-503 | Mean = 0.8%,  range: 0.02-8.1% | NA |
| SMS Logs | 201 subjects | Mean = 23,  range: 1-154 | NA | NA |
| Call Logs | 183 subjects | Mean = 14,  range: 1-78 | NA | NA |
| Ambient light | 490 subjects | Mean = 557,  range: 1-3540 | Mean = 8.9%,  range: 0.02-56.7% | NA |
| Air pressure | 50 subjects | Mean = 1180,  range: 1-5427 | Mean = 18.9%,  range: 0.02-87.0% | NA |
| Temperature | 4 subjects | Mean = 106,  range: 3-307 | Mean = 1.7%,  range: 0.05-4.9% | NA |
| Screen mode (on/off) | 569 subjects | Mean = 1220,  range: 1-6152 | Mean = 19.6%,  range: 0.02-98.6% | NA |

**S4 Table. Overview of wearable sensor data.**

|  | Chest patch | Chillband |
| --- | --- | --- |
| Nr of subjects | 924 (of which 61 do not have Chillband data) | 905 (of which 60 do not have chest patch data) |
| Nr of nights | 2979 | 1744 |
| Nr of days | 4356 | 4366 |
| Average data quality across subjects | Mean = 86.4%,  SD = 8.2% | Mean = 96.4%,  SD = 2.2% |
| Average nr of days | Mean = 4.4, range:1-6 | Mean = 4.5,  range: 1-5 |
| Average nr of hours | Mean = 97,  range: 1-184 | Mean = 107,  range: 1-130 |

**S5 Table. Overview of physiological features.**

The first column counts the features, in the second column each feature is described, the third column shows the abbreviation as how it is used further in the paper and the last column shows example references where this feature is used for stress detection.

| Nr. | Feature | Abbreviation | Reference |
| --- | --- | --- | --- |
| 1 | Mean heart rate (HR) | ECG mean HR | ^43–45^ |
| 2 | Standard deviation of RR intervals | ECG SDNN | ^11,45,46^ |
| 3 | Root mean square of successive RR differences | ECG RMSSD | ^11,46^ |
| 4 | Low frequency signal (power in the 0.04-0.015 Hz band) | ECG LF | ^11,43,44,46,47^ |
| 5 | High frequency signal (power in the 0.15-0.4 Hz band) | ECG HF | ^11,43,44,46,47^ |
| 6 | Ratio of low and high frequency | ECG LFHF | ^11,43,44,46,47^ |
| 7 | SC level – average SC | SC mean | ^45,46,48^ |
| 8 | Phasic SC – signal power of the phasic SC signal | SC phasic | ^23^ |
| 9 | SC response rate – number of SC responses (i.e. peaks) in window divided by the total length of the window (i.e. responses per second) | SC RR | ^46^ |
| 10 | SC second difference - signal power in second difference from the SC signal | SC diff2 | ^49^ |
| 11 | SC response - number of SC responses | SC R | ^10,23,45,46,48^ |
| 12 | SC magnitude - the sum of the magnitudes of SC responses | SC mag | ^10,23,45,46,48^ |
| 13 | SC duration - the sum of the duration of SC responses in seconds | SC dur | ^10,23,46,48^ |
| 14 | SC area - the sum of the area of SC responses in seconds. The area is defined using the triangular method (1/2*SC mag*SC dur) | SC area | ^48^ |
| 15 | Mean ST | ST mean | ^47^ |
| 16 | Median ST | ST median | / |
| 17 | Standard deviation ST | ST SD | ^47^ |
| 18 | Slope of the ST – slope of a straight line fitted through the data | ST slope | ^45^ |
| 19 | Standard deviation of the magnitude of accelerometer signal – a measure for movement intensity | ACC SD | ^16^ |

**S6 Table. Extended table of physiological features across self-reported stress levels.**

Population mean and 95% confidence interval (CI) of physiological features during the night (00-06 am) and different stress levels (S1, S2 and S3).

|  | Night (mean, 95% CI) | S1 (mean, 95% CI) | S2 (mean, 95% CI) | S3 (mean, 95% CI) |
| --- | --- | --- | --- | --- |
| ECG mean HR | 62.5 (47.3, 83.3) | 72.1 (52.1,95.1) | 73.4 (53.6, 95.5) | 74.6 (56.0, 96.1) |
| ECG SDNN | 74.6 (21.0, 171) | 69.9 (24.2, 141.7) | 70.6 (27.0, 136.8) | 69.4 (27.5, 129.4) |
| ECG RMSSD | 985 (722, 1273) | 856 (634, 1157) | 839 (631, 1123) | 824 (627, 1076) |
| ECG HF (x10^-3^) | 0.69 (0.018, 3.6) | 0.62 (0.016, 2.9) | 0.66 (0.024, 2.8) | 0.62 (0.023, 2.7) |
| ECG LF (x10^-3^) | 1.1 (0.054, 5.0) | 1.1 (0.074, 4.2) | 1.1 (0.097, 4.0) | 1.1 (0.1, 4.0) |
| ECG LFHF | 3.5 (0.29,14.6) | 3.8 (0.48, 15.0) | 3.5 (0.51, 13.8) | 3.7 (0.53, 14.4) |
| SC mean | 2.6 (0.008, 12.5) | 1.4 (0.030, 8.8) | 1.4 (0.024, 8.5) | 1.5 (0.025, 8.3) |
| SC phasic | 18.3 (0, 157.5) | 8.3 (0, 78.9) | 7.7 (0, 72.5) | 8.2 (0, 70.4) |
| SC RR (x10^-2^) | 2.7 (0, 14.0) | 2.5 (0, 12.6) | 2.7 (0, 12.3) | 2.9 (0, 13.0) |
| SC diff2 (x10^-9^) | 20.7 (0, 163) | 30.0 (0, 214) | 25.7 (0, 189) | 38.9 (0, 239) |
| SC R | 3.9 (0, 25) | 12.9 (0, 91) | 12.5 (0, 87) | 14.0 (0, 98) |
| SC mag | 44.6 (0, 392) | 123 (0, 1050) | 111 (0, 947) | 129 (0, 1094) |
| SC dur | 355 (0, 2508) | 1274 (0, 8691) | 1248 (0, 8446) | 1363 (0, 9255) |
| SC area | 0.71 (0, 6.2) | 1.9 (0, 16.0) | 1.7 (0, 14.5) | 2.0 (0, 16.5) |
| ST mean | 32.9 (21.0, 36.0) | 31.5 (27.3, 35.0) | 31.2 (27.1, 34.0) | 31.2 (27.6, 34.0) |
| ST median | 32.9 (21.0, 36.0) | 31.5 (27.0, 35.0) | 31.2 (27.0, 34.0) | 31.3 (28.0, 34.0) |
| ST SD | 0.10 (0, 0.50) | 0.14 (0, 0.50) | 0.13 (0, 0.50) | 0.13 (0, 0.50) |
| ST slope (x10^-3^) | 0.061 (-4.6, 4.7) | 0.34 (-4.4, 4.9) | 0.30 (-4.3, 4.9) | 0.31 (-4.2, 4.9) |
